# Hoffmann’s Syndrome Secondary to Pendred Syndrome: A Rare Case

**DOI:** 10.7759/cureus.4195

**Published:** 2019-03-06

**Authors:** Faryal Tahir, Laila Tul Qadar, Maria Khan, Hareem Hussain, Syed Umair Iqbal

**Affiliations:** 1 Internal Medicine, Dow University of Health Sciences, Karachi, PAK; 2 Surgery, Dow University of Health Sciences, Karachi, PAK

**Keywords:** hoffmann syndrome, pendred syndrome, hypothyroidism, pseudohypertrophy

## Abstract

Hoffmann’s syndrome (HS) is a rare manifestation of hypothyroidism myopathy that presents with weakness, stiffness, and eventually pseudohypertrophy of muscles, especially calf muscles. We report a case of a 28-year-old male who presented with the history of generalized weakness with swelling in lower limbs and gradual progressive facial puffiness for the past few years. Physical examination of our patient showed diffuse bilateral pseudohypertrophy of deltoid and calf muscles with positive Gowers’ sign (GS). Laboratory results of low serum thyroid hormones and muscle biopsy report confirmed the diagnosis of HS. Pendred syndrome (PS) is a genetic disorder leading to congenital bilateral sensorineural hearing loss with mild hypothyroidism. On account of his congenital bilateral sensorineural hearing loss and negative serum anti-thyroid peroxidase antibodies (anti-TPO Ab), PS was declared as the cause of HS in this case. Our patient showed excellent response to levothyroxine therapy with progressive improvement in his symptoms. We outlined this case due to its rarity.

## Introduction

A diverse array of presentations of hypothyroidism is seen in routine clinical practice. The symptoms of patients with hypothyroidism vary from mild fatigue to fatal myxedema coma [1]. The incidence of myopathy in hypothyroidism ranges from 30% to 80%. The main symptoms related are weakness, muscle cramps, and myalgia [2]. Thyroid hormone has considerable effects on every system of the human body. An abnormality in thyroid hormone levels (THLs) results in the fluctuation of normal metabolism of the body, therefore, leading to a myriad of abnormalities. Low serum THLs lead to abnormal changes in muscle tissue. Myopathy associated with hypothyroidism can be categorized into four subtypes: (1) Kocher-Debre-Semelaigne syndrome (KDSS), (2) Hoffmann’s syndrome (HS), (3) atrophic form, and (4) myasthenic syndrome (MS) [3]. Hypothyroidism rarely affects adults as HS. It is described as the presence of hypothyroidism along with pseudohypertrophy of certain muscles and muscular weakness [4]. Along with decreased serum THLs and raised serum thyroid stimulating hormone (TSH), elevation in muscle enzymes is also observed in HS indicating rhabdomyolysis [5]. Here, we report a rare case of HS in a 28-year-old male secondary to Pendred syndrome (PS) which was effectively managed with thyroid hormone replacement therapy (THRT).

## Case presentation

**Figure 1:**
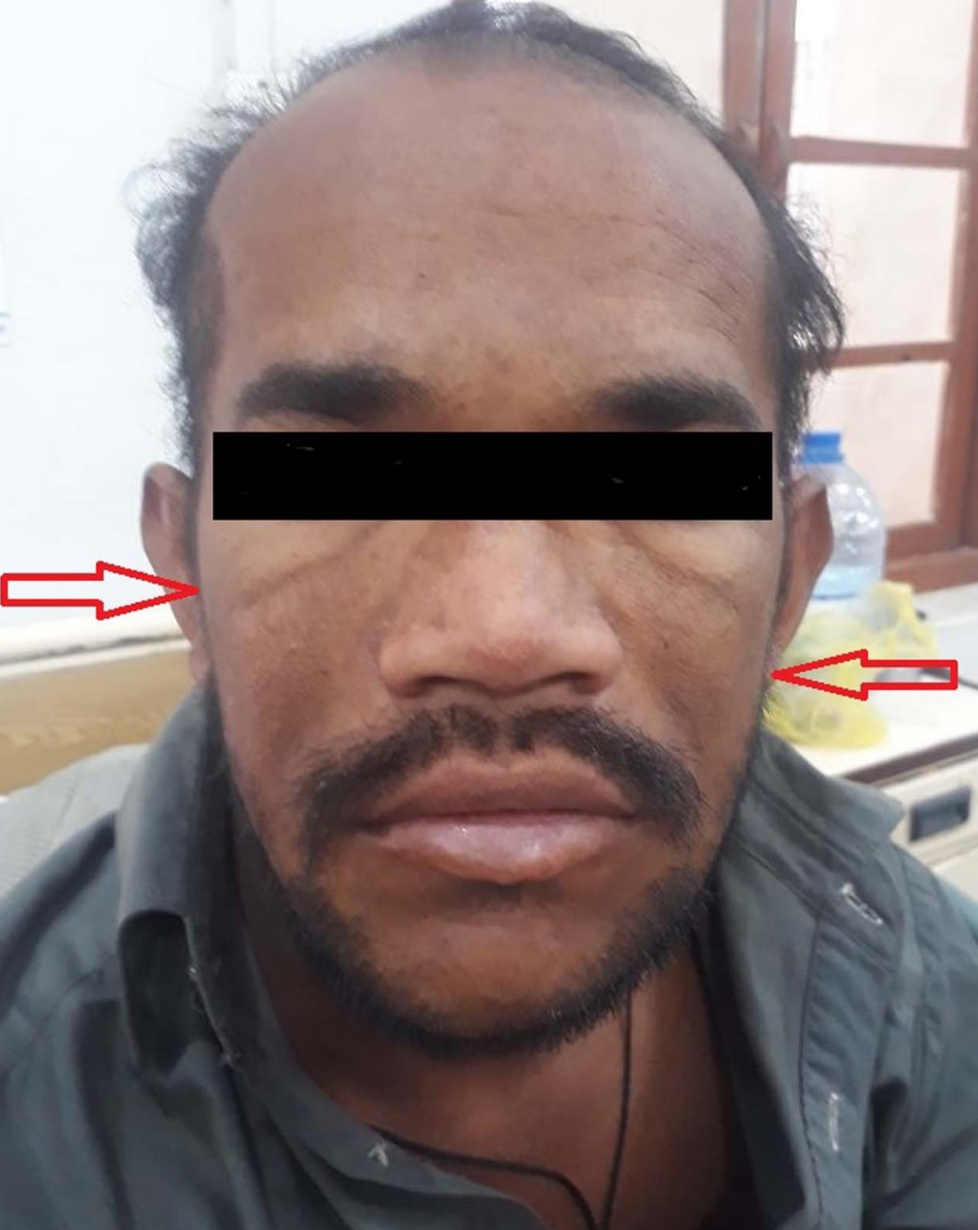
Facial puffiness of hypothyroid patient suffering from Hoffmann's syndrome.

A 28-year-old male, deaf and dumb since birth, presented to the medical department (MD) of Dr. Ruth KM Pfau, Civil Hospital Karachi (CHK) in November 2018 for the evaluation of generalized upper and lower limb weakness along with progressive facial puffiness (Figure 1) for the past nine years. There was also a history of lower limb swelling for the past seven years. 

**Figure 2:**
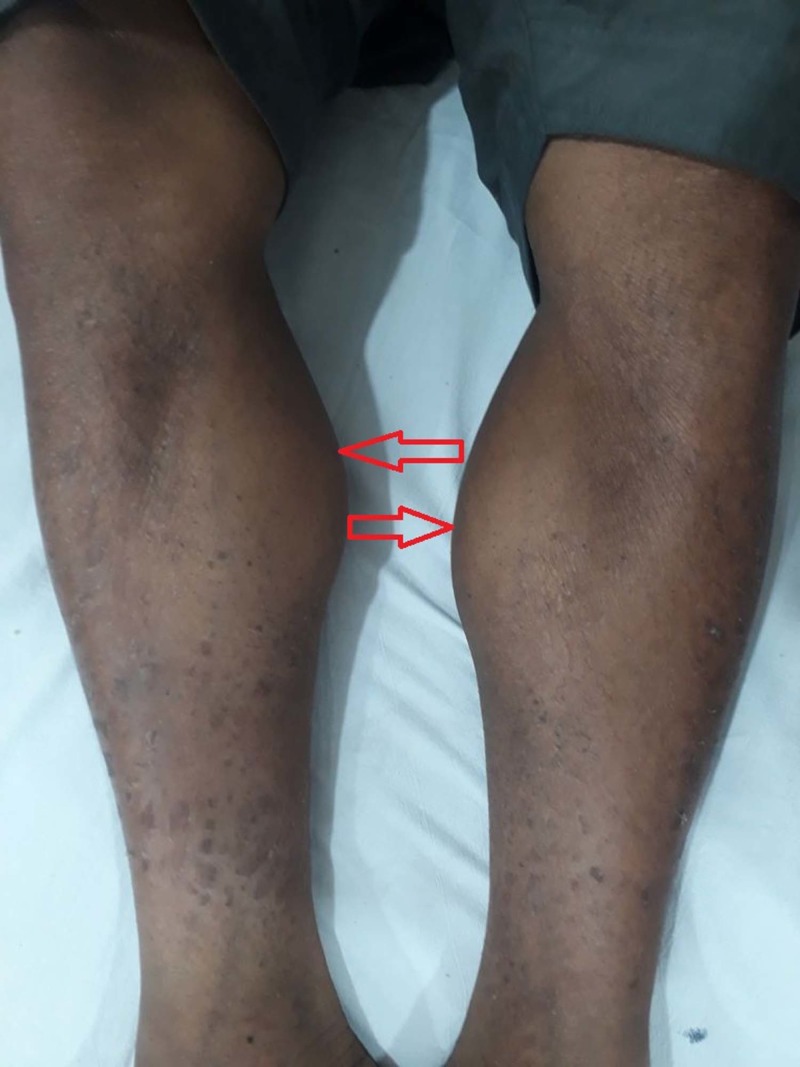
Bilateral pseudohypertrophy of calf muscles.

On examination (O/E), his pulse was 82 beats/min, blood pressure (BP) was 110/70, respiratory rate (RR) was 16 breaths/min, and he was afebrile. There was no thyroid enlargement. Lower limbs showed nonpitting edema up to the ankles. Upon neurological examination, bulk showed diffuse bilateral pseudohypertrophy of the calf muscles (Figure 2) and the deltoids, tone was normal, power was 4/5, reflexes were 2+, and Gowers’ sign (GS) was positive. The skin was coarse and dry with dermopathy. Systemic examination was normal.

Laboratory investigations revealed hemoglobin (Hb) of 11.7 gm/dl, raised serum cholesterol of 310 mg/dl, raised TSH of 35.2 μIU/ml [Normal (N) = 0.4-4.0], decreased levels of thyroxine (T4) of 0.14 μg/dl (N = 4.5-12.5), low plasma triido thyronine (T3) of 26.2 ng/dl (N = 84-172), anti-thyroid peroxidase antibody (anti-TPO Ab) <10 (N = <35), elevated creatine phosphokinase (CPK) of 1108 U/L (N= 85-170), creatine kinase-muscle and brain (CK-MB) of 126 U/L, elevated lactate dehydrogenase (LDH) of 930 U/L (N = 225-450), and raised serum aldolase of 9.3 U/L (N = 0-7). Blood glucose, liver function tests, blood urea, and serum creatinine were not remarkable. Abdominal X-ray revealed bilateral renal cortical cysts; however, no calculus or hydronephrosis was seen in both kidneys. Evaluation of cardiovascular and respiratory systems did not show any significant abnormality. Electromyography (EMG) and nerve conduction study (NCS) were normal. A biopsy was taken from his right deltoid muscle which showed mild changes in fiber size and local fibrosis. The findings were suggestive of myopathy. Immunohistochemistry was not done due to financial constraints. 

A diagnosis of HS was made based on clinical manifestations, laboratory investigations, and confirmatory muscle biopsy report. We further suspected PS as the cause of hypothyroidism in this patient based on his symptoms of congenital deafness and thyroid dysfunction. A tone audiometry was carried out which showed bilateral sensorineural hearing loss. A diagnosis of PS was finally established by performing a perchlorate discharge test which showed an abnormally rapid loss of radioactive iodine from the thyroid gland after the administration of perchlorate.

The patient was initially treated with 100 μg levothyroxine daily which was gradually increased to 150 μg after two weeks. The patient was assessed after four weeks. His swelling resolved and TSH levels went down to 13 μIU/ml . The patient is under regular follow up with improvements in his symptoms.

## Discussion

In 1897, HS was first elucidated by Hoffmann in an adult with muscle stiffness and hindrance in relaxation of his muscles following thyroidectomy [6]. This suggests hypothyroidism as one cause of myopathy. The most common myopathic signs and symptoms observed in hypothyroidism include weakness of proximal muscles with cramps, myxedema on percussion with delayed deep tendon reflexes, and rarely, muscle hypertrophy [7]. It is quite uncommon for muscle hypertrophy to be the initial presentation of hypothyroidism with minimal systemic features [8] as seen in this case. Although this syndrome can affect any muscle, the predominantly involved muscles are arm, thigh, leg, and tongue while gastrocnemius showing hypertrophy most of the times [9]. This patient showed decline in the level of thyroid hormones and an elevated TSH levels with associated bilateral pseudohypertrophy of calf muscles, so a diagnosis of HS was made.

Usually primary hypothyroidism leads to thyroid insufficiency, Hashimoto's thyroiditis being the main culprit. Contrary to this, a suspicion of PS as the cause of hypothyroidism was made in this case because of the associated sensorineural hearing loss since birth along with the negative serology for anti-TPO Ab. Immunohistochemistry could not be done due to the nonavailability of funds.

Levels of CPK, being the best biochemical indicator of myopathies [10], were dramatically increased in this patient. In the absence of liver disease, other enzymes such as aldolase and LDH have a correlative role [10] and were also elevated in this patient. Treatment ensues a fall in muscle enzymes along with TSH levels and can take a variable course, occurring over a period of weeks, months or in some cases, even years [11-12]. In the presented case, muscular enzymes and TSH levels decreased dramatically by the time the patient was discharged and are hoped to have normalized in the next few follow ups.

Patients are often misdiagnosed as the clinical picture and biochemical studies might challenge the physician to differentiate this syndrome from polymyositis or muscle dystrophies. EMG, NCS, and muscle biopsy are useful tools for the establishment of diagnosis. In this case, EMG and NCS were normal. Muscle biopsy revealed pale muscle fibers with variation in their size and focal areas of fibrosis without any inflammatory infiltrate, excluding polymyositis. This patient experienced difficulty in walking presumably because of frail contractions of the involved muscles. This weakness of muscular contraction associated with hypothyroidism is caused by muscle fiber transformation from type 2 fast-twitching to type 1 slow-twitching and abnormal oxidative enzymatic function [9]. The exact cause of hypertrophy of muscle remains enigmatic, but it is thought to be due to an upsurge in the size and number of muscle fibers as well as the amount of connective tissue [2, 9]. Muscle hypertrophy can also be a result of accumulated glycosaminoglycans (GAGs) [9]. All these findings with elevated TSH level faded the suspicion of muscle dystrophies. This proves that screening of these patients with simple thyroid profile can help in making a definitive diagnosis.

The treatment comprises exogenous thyroid hormone, in the form of levothyroxine, the dosage being 100-200 µg/day. Cardiovascular status of this patient was found to be normal as it is vital to rule out any cardiovascular risks prior to starting treatment due to an increased risk of acute coronary insufficiency, more common in elderly. A typical surge in symptoms can be seen in some cases with severe myopathic manifestation after the initiation of treatment, possibly due to raised metabolic demand induced by thyroxine [3]. In these cases, the adjuvant use of corticosteroids is recommended due to their membrane-stabilizing effect. However, this patient responded well to THRT as a sole therapy.

## Conclusions

Hoffmann’s syndrome is a rare clinical presentation of a very common disorder of endocrine system, hypothyroidism. This case is even more rarer as it indicates PS as one cause of hypothyroidism leading to HS if left untreated. The positive aspect of this unusual entity is that it carries favorable prognosis with complete reversal of symptoms on timely made diagnosis followed by prompt initiation of THRT. As in this case, the patient showed gradual but progressive recovery from myopathy and reduction in elevated levels of TSH and muscle enzymes. Clinicians should be well aware of this rare manifestation of hypothyroidism to prevent the associated morbidity by avoiding misdiagnosis and subsequent delay in management.
